# Autism Pathogenesis: The Superior Colliculus

**DOI:** 10.3389/fnins.2018.01029

**Published:** 2019-01-09

**Authors:** Rubin Jure

**Affiliations:** Centro Privado de Neurología y Neuropsicología Infanto Juvenil WERNICKE, Córdoba, Argentina

**Keywords:** autism spectrum disorders (ASD), autism pathogenesis, human development, congenital blindness, pulvinar, visual pathways, superior colliculus (SC)

## Abstract

After been exposed to the visual input, in the first year of life, the brain experiences subtle but massive changes apparently crucial for communicative/emotional and social human development. Its lack could be the explanation of the very high prevalence of autism in children with total congenital blindness. The present theory postulates that the superior colliculus is the key structure for such changes for several reasons: it dominates visual behavior during the first months of life; it is ready at birth for complex visual tasks; it has a significant influence on several hemispheric regions; it is the main brain hub that permanently integrates visual and non-visual, external and internal information (bottom–up and top–down respectively); and it owns the enigmatic ability to take non-conscious decisions about where to focus attention. It is also a sentinel that triggers the subcortical mechanisms which drive *social motivation* to follow faces from birth and to react automatically to emotional stimuli. Through indirect connections it also activates simultaneously several cortical structures necessary to develop *social cognition* and to accomplish the multiattentional task required for conscious social interaction in real life settings. Genetic or non-genetic prenatal or early postnatal factors could disrupt the SC functions resulting in autism. The timing of postnatal biological disruption matches the timing of clinical autism manifestations. Astonishing coincidences between etiologies, clinical manifestations, cognitive and pathogenic autism theories on one side and SC functions on the other are disclosed in this review. Although the visual system dependent of the SC is usually considered as accessory of the LGN canonical pathway, its imprinting gives the brain a qualitatively specific functions not supplied by any other brain structure.

## Introduction

**FIGURE 1:**
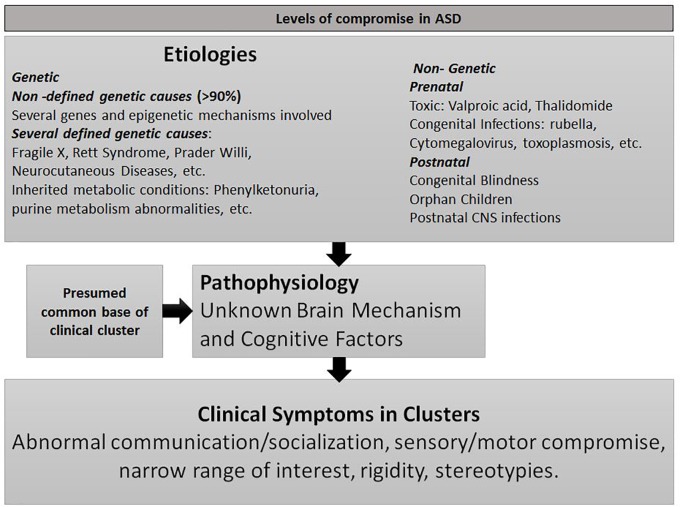
Levels of compromise in ASD.

Autism spectrum disorder (ASD) is not a disease; it is a syndrome with hundreds of genetics and non-genetics etiologies (see Figure 1) and with broad clinical manifestations. Its pathogenesis, scarcely known, is also presumed to be heterogeneous (Waterhouse et al., 2016). The coherence of the syndrome lies in the presence of the core symptoms in *cluster* (Hobson, 2014): ASD individuals with extremely varied etiologies, cognitive levels, and cultures share a broad range of specific symptoms in social, communicative, cognitive, motor and sensitive domains. This strongly suggests that diverse pathogenic mechanisms could have a common final pathway which, in turn, compromises several brain networks resulting in disparate symptoms. The unsuccessful efforts to find it could be due to the limitations of *in vivo* investigation on the developing human brain (Courchesne et al., 2007) and to the fact that social cognition and communication are mainly non-consciously acquired during early life. Hence, little is known about the functional and cognitive processes that govern its development.

An often overlooked fact that could be a clue for pathogenic research is that *a complete lack of vision during the first year of life results very frequently in a full-blown ASD syndrome*. During early life, visual input is dominated by the SC and the Pul (Bridge et al., 2015). The SC is an extremely ancient and complex subcortical structure, not only essential for simultaneous visual attention and eye movements as it is usually believed. It also has a crucial role in the integration of external and internal senses with emotional, autonomic and endocrine functions, as well as in visual/motor transformation, target selection and goal-directed motor responses (Merker, 2007; Krauzlis et al., 2013). It is also the neurologic substrate of *innate* behaviors (Furigo et al., 2010) like automatic attention to faces and biologic movement (Nguyen et al., 2014; Rosa Salva et al., 2015; Mares et al., 2016), social motivation, and innate fear (e.g., to snakes) (Liddell et al., 2005; Wei et al., 2015). Due to its strategic location as a first line visual and multisensory structure to receive environmental input and its multiple *direct* connections from the cortex, the SC is in the interphase of several complex processes: (1) the bottom–up and top–down attentional interplay (Merker, 2013), (2) the shift from covert to overt attention (Sato et al., 2016), and (3) the interaction of medial and lateral cortical brain networks that regulate and integrate endogenous with externally drive attention respectively (Menon and Uddin, 2010; Katyal and Ress, 2014; Mysore and Knudsen, 2014; Xuan et al., 2016). These dimensions are essential not only to process ongoing information but also to mental time travel, to mentalize abilities (Corbetta et al., 2008) and to develop complex representations of the self (Fabbro et al., 2015). But, perhaps, the most relevant aspect for ASD pathogenesis is the evidence that the SC has a plastic role in the postnatal microstructure of the brain in an exclusive way that favors social/communicative abilities (Maior et al., 2012; Rosa Salva et al., 2015; Soares et al., 2017).

The aim of this work is to show that different lines of research on ASD etiologies and pathogenesis, including cognitive theories, functional studies and anatomic evidence converge on the SC.

## ASD and Congenital Blindness

From the first report of autism in blind children made 60 years ago (Keeler, 1956), a review of 12 different studies yields a ∼50% of prevalence of ASD in early blindness (Jure et al., 2016). The prevalence is even higher when only children with total CB are considered. In a school for the blind, ASD was found in 18 of 25 (72%) students with CB and in only 1 of 13 (8%) with partial or acquired blindness. Statistical analysis showed that CB was the main responsible factor. No other variable, such as etiology of blindness or socioeconomic family status, accounted for this very high ASD prevalence (Jure et al., 2016).

Another similarity lies in the evolution of symptoms; besides the most common chronic clinical course of ASD, two other subgroups have been described in children with CB: one that showed an early *autism regression* and a second one with a late *autism recovery*. Both occur at the same age window as in the sighted: *regression* takes place between 15 and 30 months (Cass et al., 1994; Rosenbloom, 1994; Dale and Sonksen, 2002; Luyster et al., 2005; Jure et al., 2016) and *recovery* occurs beyond 10 years in sighted/ASD (Fein et al., 2013) and CB/ASD individuals (Hobson and Lee, 2010; Jure et al., 2016). However, *regression* is ∼3000 times more frequent in CB than in the sighted (Dale, 2005) and *recovery* in CB was not only limited to high functioning ASD individuals (Hobson and Lee, 2010; Jure et al., 2016) as it was described in sighted ASD subjects (Fein et al., 2013).

The high prevalence of autism in early blindness has been overlooked until the present (Hobson and Bishop, 2003; Tager-Flusberg, 2005). Several reasons might explain this lack of attention (Jure et al., 2016). Perhaps, the strongest is the automatic assumption that it is not a “true” autism because it is secondary to blindness. However, autism is *exclusively* defined clinically (DSM or ICD criteria). Comparatively, a similar clinical syndrome (e.g., spastic hemiparesis) in different individuals usually reflects similar brain compromise (pyramidal tract), independent of the etiology (cerebral infarct, brain malformation, etc.). To exclude CB as one etiologic factor of ASD would be similar to exclude tuberous sclerosis, fragile X, fetal valproate syndrome as well as hundreds of other ASD etiologies.

Diffuse postnatal brain changes occur during the first year of life by visual influence and the microstructure and functionality of the brain is different in CB, resulting in atypical neurodevelopment (Coullon et al., 2015; Hasson et al., 2016; Reislev et al., 2017). Humans studies point to the SC as the main structure responsible of those changes (Coullon et al., 2015).

## The Role of Vision in Development

Visual input has a pervasive influence on sensorimotor coherence, cognition, behavior and social development and this is an area of active research (Itier and Batty, 2009; Young et al., 2009; Graham and LaBar, 2012; Landgraf and Osterheider, 2013; Leigh and Zee, 2015; Zihl and Dutton, 2015). The brain is almost continuously and automatically creating a visual representation of the world in a self-centered perspective (Merker, 2013; Zihl and Dutton, 2015). Non-conscious integration of vision with somatosensory input allows the differentiation between the self and the environment (Fraiberg, 1977). After birth, vision is the main tool to understand the meaning of the surrounding world including objects and people, the notion of object permanence, and the cause-effect relationships that govern actions.

Social development is the result of an interaction between innate forces -*social motivation*-, and late developed abilities environmentally influenced -*social cognition*. These behaviors are supported by extensive but selective subcortico/cortical brain networks named “the social brain network” (Senju and Johnson, 2009b; Graham and LaBar, 2012; Mares et al., 2016) which is automatically activated when a face enters in our visual field (Elgar and Campbell, 2001; Fletcher-Watson et al., 2008; Klin et al., 2015). At 10 min of life the visual attention of the baby is not random. Innate forces govern an attraction for tracking *faces* or face-like patterns (Goren et al., 1975). This behavior declines at the end of the first month to enable a more developed face processing (Johnson et al., 1991), but a strong bias for ultra-rapid automatic detection of faces remains lifelong (Fletcher-Watson et al., 2008). The child also pays special attention to *human motion* and *visual communication* (Klin et al., 2015). Non-verbal pragmatics like eye contact, reciprocal smiling, and taking turns in sound exchanges are one of the first behaviors to emerge, and the earliest mouth movements to produce sounds are led by vision (Skipper, 2014). Attention to the mouth predicts larger vocabulary size in normal children (Young et al., 2009) and infants frequently focus on the speaker’s mouth (Lewkowicz and Hansen-Tift, 2012). *Gaze direction* is highly informative of the other’s intention and is pivotal for *Joint Attention*, essential to encode a new label for an object. The eyes are the most attended feature of the face as they give several fundamental clues for surviving and well-being: emotion, gender and identity recognition (Itier and Batty, 2009). Some authors proposed that this innate eye and face detector system (Baron-Cohen, 1997; Itier and Batty, 2009) is more sensitive to emotional expressions than identity or gender features (Lundqvist et al., 2014; Neath and Itier, 2015; Palanica and Itier, 2015; Neath-Tavares and Itier, 2016). Abnormalities in *visual attention* and *gaze behavior* are universally present in the ASD population and have been highlighted in its first description by Kanner (1943). Growing evidence suggests that automatic, non-conscious attention to faces, eyes and biologic movement is mediated by the SC-Pul-Amy complex (Senju and Johnson, 2009a; Tamietto and de Gelder, 2010; Campatelli et al., 2013).

Full communication in real life settings requires the integration of several verbal and visual features that are processed separately (Bolis and Schilbach, 2017). Mother/infant social-motor body and emotional synchrony have an important influence on the development of intersubjectivity, social and language learning. “Interpersonal synchronization” in time and content encompasses joined attention, imitation (Marsh et al., 2013), turn-taking, non-verbal social communicative exchanges, affect sharing and engagement (Brenner et al., 2007; Zhao et al., 2017).

Regarding postnatal visual influence on brain development, it was proposed that the subcortical SC-Pul-Amy complex “tutors” slow developing cortical networks, allowing further specialization on social abilities (Johnson et al., 2015). Indirect evidence and animal studies suggest that early social visual experiences have an epigenetic influence on subcortical *experience-expectant* visual circuits, turning on and off specific genes resulting in plastic effects on *experience-dependent* subcortico-cortical networks conforming the *social brain* (Johnson et al., 2005; Klin et al., 2015; Rosa Salva et al., 2015). The microscopic changes that occur during the first months of life are perhaps the epitome of the complexity of the interactions between genetic and epigenetic environmental factors necessary to shape the human brain. The peak of synaptic growth is reached approximately at 12 months old, followed by a widespread pruning in early childhood; long-range brain connections increase from birth to early adulthood (Vértes and Bullmore, 2015).

## Two Visual Pathways

**FIGURE 2:**
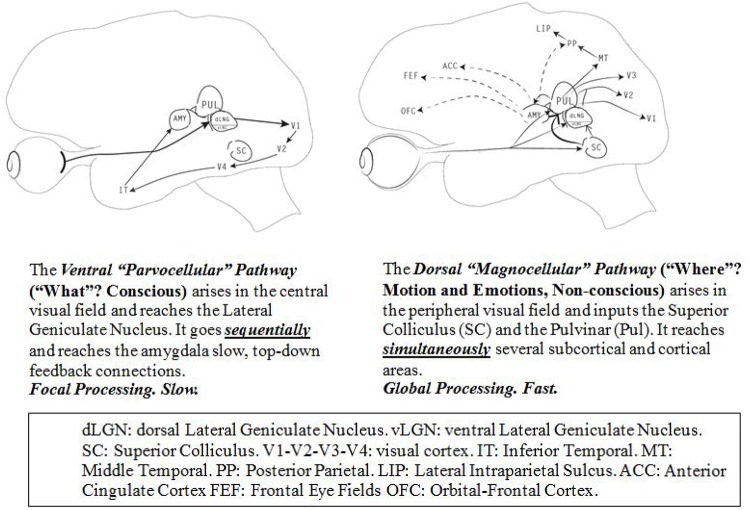
Two visual pathways.

Although we perceive vision as a unitary function, there are two visual pathways with different but highly complementary and interconnected functions (Figure 2). The classic *ventral pathway* receives *parvocellular* input from numerous small cones, which are responsible for the central, foveal retinal vision. It represents ∼90% of the visual input, and it is unifocal and slower than the dorsal pathway. It allows a conscious, fine resolution, and a detailed and colorful analysis of the stimuli. After reaching the primary visual striate occipital cortex (V1) directly through the dorsal lateral geniculate nucleus (dLGN), it goes mainly *sequentially* through the extrastriate cortex (V2-V4) up to the infero-temporal area. Any object or event that is under conscious scrutiny occupies the central vision and flows through this pathway.

The *dorsal pathway*, extremely fast but of low resolution, receives *magnocellular* and koniocellular input from the rods, located in the peripheral visual field. It is *multifocal*, mainly non-conscious or pre-attentive and very sensitive to any movement or change in contrast luminescence. It allows recognition of *where* things are located in space as a result of the permanent input from both visual fields; its main first stations are the SC, the Pul and the vLGN. It reaches *simultaneously*, the extrastriate (V2-V3), the striate visual cortex (V1), and the middle-temporal cortex (MT). This last structure provides ongoing information about the visual environment to the posterior parietal (PP) and the LIP, essential to register sequences of movements (Lamme and Roelfsema, 2000).

Although both networks interact and contribute to non-conscious and conscious vision (Breitmeyer, 2014), the dorsal pathway is by far the most prevalent for pre-attentive and non-conscious processing as it is faster and it is continuously screening the peripheral visual field through the SC, a brain sentinel specially trained to select any biological relevant information such as *motion, emotions and novelty* (Tamietto and de Gelder, 2010; Tamietto, 2011; Soares et al., 2017). Most evidence indicates that the dorsal pathway is responsible for the visual behavior in the first months of life (Hammarrenger et al., 2003; Johnson, 2005; Bridge et al., 2015).

## Blindsight

*Blindsight* is the ability to accurately use visual information without involvement of the primary visual cortex (V1), in absence of conscious visual perception (PöPpel et al., 1973; Weiskrantz et al., 1995). It is not a unitary phenomenon: *“attentional blindsight”* refers to the ability to make a visual discrimination in the blind hemifield, while *“action blindsight”* is the ability to respond with saccades, smooth eye pursuits, pointing or grasping to a new target or to a moving stimulus present in the blind hemifield (Danckert and Rossetti, 2005), specially to looming images (Hervais-Adelman et al., 2015). A surprising finding was the demonstration of *“emotional blindsight”* (de Gelder et al., 1999) that denotes the non-conscious capacity to correctly respond to emotional salient visual stimuli as fear, happy or angry faces, or whole-body emotional expressions in the blind hemifield (Van den Stock et al., 2011; Celeghin et al., 2015; Diano et al., 2017). These findings strongly suggest the existence of automatic, non-conscious activation of several input-processing-output subcortical circuits (SC-Pul-Amy) of the dorsal visual pathway (Tamietto and de Gelder, 2010; Diano et al., 2017; Soares et al., 2017). Based on animal and human studies, most authors maintain that *blindsight is exclusively mediated by the SC* (Leh, 2006; Leh et al., 2006; Tamietto et al., 2010), while a few claim that it is driven by the LGN (Breitmeyer, 2014; Ajina et al., 2015). The most convincing evidence of the exclusivity of the SC on blindsight is that a *selective* inactivation of the visual SCs in monkeys severely compromised non-conscious guided behavior (Kato et al., 2011).

Through the SC the dorsal pathway reaches several alternative (non-canonical) visual structures simultaneously allowing a panoramic processing of space with special sensitivity to faces, eyes, biological movement and emotional clues. This *fast detection* of relevant stimuli (Soares et al., 2017) is followed by a *selection*, also provided by the SC, which results in a shift from peripheral to central vision up to five times in a second if necessary (orienting attention) (Fecteau and Munoz, 2006).

## Previous ASD Theories

Visual abilities are superior to language in most ASD individuals; hence, it seems paradoxical to look for a pathogenic theory based on the high prevalence of ASD in CB. Nevertheless, as it will be exposed in this section, most of the current ASD pathogenic theories are based on visual processing and visual attention.

### Magnocellular Pathway Dysfunction Theories

Compromise on motion perception in ASD individuals (Gepner et al., 1995) and abnormal face processing from early life to adulthood has been proposed to be secondary to magnocellular dysfunction (Milne et al., 2002; McCleery et al., 2007). A *direct* relationship has been found in the degree of compromise on peripheral vision secondary to dysfunctions on the magnocellular pathway and the degree of autism severity (Sutherland and Crewther, 2010; Crewther and Crewther, 2014; Crewther et al., 2015; Vanmarcke and Wagemans, 2016). Greenaway et al. (2013) remark that previous evidence against selective and marked compromise of the magnocellular pathway in ASD individuals is the result of methodological issues in assessment. The only histological evidence of abnormal sensory input in ASD is the description of complete lack of normal magnocellular neurons on the LGN in Fragile X/ASD individuals (Kogan, 2003). Unfortunately, this excellent work did not include SC magnocellular investigation.

### Global vs. Local Visual Perception Theories

The WCC was initially proposed as a diminished ability in the global top–down processing, essential for the integration of detailed features in a coherent whole, to explain social and non-social deficits in ASD individuals (Frith and Happé, 1994). Years later the same authors suggested that instead of a deficit in global processing, a *bias* for local processing resulted in superiority in visual perception of details (Happé and Frith, 2006). Similarly, the authors of a related theory – the EPF – (Mottron et al., 1999; Mottron et al., 2006) that initially postulated a stronger local processing in ASD individuals, then suggested an automatic *attention bias* for local visual information (Guy et al., 2016). This is in consonance with an extensive meta-analysis review showing that typical individuals perceived automatically the whole picture before conscious attention to details, a pattern that seems to be reversed in ASD (Van der Hallen et al., 2015). Additionally, drawing tasks comparing typical children with those with ASD did not show better local performance supporting the theory of *bias* on visual attention (Smith et al., 2016).

### Innate Motivation to Social Attention

There is a growing body of research focusing on innate motivational aspects that *bias* social learning in an atypical way (Dawson et al., 2004). The *Social Motivation theory of ASD* focuses on a prenatal compromise in the *experience expectant* circuits (Chevallier et al., 2012) prepared to attend to faces and biologic stimuli as soon as the child is born (Klin et al., 2015; Rosa Salva et al., 2015). Both, lack of social motivation as a result of hypoarousal, or an excess of negative feelings provoked by hyperarousal states have been proposed to explain ASD social abnormalities (Senju and Johnson, 2009a; Chevallier et al., 2012). Emerging evidence seems to support this approach, at least in a subgroup of ASD individuals (Gamer et al., 2010; Assaf et al., 2013; Skuse et al., 2014).

### Face Processing

Face and gaze processing has been one of the most active research topic in ASD for its prevalent role on socialization and communication and due to the observance of its abnormal developmental pattern in autistic children (Elgar and Campbell, 2001; Senju and Johnson, 2009a; Senju et al., 2011; Elsabbagh et al., 2012; Weigelt et al., 2012; Jones and Klin, 2013). After reviewing extensive behavioral and neurophysiologic studies on ASD individuals, Senju and Johnson proposed a “fast track modulator model” theory based on an early dysfunction in the subcortical face and eye contact detection route: *the SC-Pul-Amy complex* (Senju and Johnson, 2009a; Senju et al., 2011). Similarly, Campatelli et al. (2013) remark the existence of weakly coordinated interacting networks for normal face processing in ASD*: the subcortical “face detection network” (SC-Pul-Amy)* activates the “gaze/action representation network” (STS, sensori-motor cortex and IFG), the “emotional evaluation network” (Amy, insula and limbic system) and the “face identification network” (fusiform gyrus and inferior occipital gyrus). MEG images corroborate that normal face processing is mediated by *an automatic visual pathway* that triggers diverse extra-striate cortical visual areas, a pattern of activation that is different in ASD individuals (Bailey et al., 2005). Human fMRI studies also suggest a dysfunction on the *automatic face detection subcortical system involving the SC-Pul-Amy* in autistic individuals (Kleinhans et al., 2011), or an exaggerated subcortical activation that results in eye avoidance (Hadjikhani et al., 2017).

While some authors described that ASD individuals actively avoid looking at fearful faces (Corden et al., 2008), others found a direct relationship between autism severity and lack of attention to happy faces (Lassalle and Itier, 2015). Contradictory results could be explained by basic attentional factors such as the “arousal effect”: emotional potency or perceptual saliency (Lundqvist et al., 2014). A compromise of the normal attentional early orienting *bias* for emotional faces was significantly related to autism severity and *not* with ADHD and anxiety symptoms in children (Antezana et al., 2016).

A clinical study of high risk population infants revealed that the earlier manifestations of ASD were abnormalities in eye contact, visual tracking and visual attention (Zwaigenbaum et al., 2005). Neurophysiological studies showed abnormal sensitivity to dynamic eye gaze and atypical patterns of processing faces in those that later developed ASD (Elsabbagh et al., 2012; Keehn et al., 2015). An *abnormal curve of development in eye contact* was found in a prospective follow up study on infants later diagnosed as autistic (Jones and Klin, 2013). Klin et al. (2015) found that toddlers with ASD fail to recognize and to orient to *biological motion perception*, a skill present in newborns and even in lower vertebrates like hatched chicks. They suggest focusing future research on children between 4 and 12 weeks of life, when the transition from subcortical to cortical control seems to occur.

### Attention in ASD

Described from the beginning (Kanner, 1943), attentional abnormalities are usually presented as associated symptoms of ASD. Instead, there is a renewed interest in the initial proposal of the role of early attention and its disruption as a core dysfunction with seminal influence on ASD symptomatology (Gold and Gold, 1975). *According to different authors, even a slight compromise on basic attentional processing on early life could have an exponential effect on joint attention, affecting language and social development* (Zwaigenbaum et al., 2005; Mundy and Newell, 2007; Chawarska et al., 2013; Elsabbagh et al., 2013; Sacrey et al., 2014) *overfocusing, restrictive range of interest and repetitive behaviors* (Keehn et al., 2013; Stevenson et al., 2014). It was found that attention to the speaker’s mouth and eyes influenced the development of language in typical and ASD children (Young et al., 2009), but not in children with language disorders, *suggesting that the language compromise on children with autism may be driven in part by abnormal social attention* (Tenenbaum et al., 2014). Longitudinal studies of ASD children showed beneficial effects of early stimulation of joint attention on verbal language development (Gulsrud et al., 2014).

#### Social and Non-social Attentional Compromise

Impairments in *shifting attention* to novel visual and auditory stimuli have been proposed as the basic compromise on ASD’ individuals by Courchesne et al. (1985); they point to the cerebellum as responsible for these abnormalities (Courchesne et al., 1994; Fatemi et al., 2012). A general decreased spontaneous visual attention to *both*, faces and objects, has been found in autistic individuals of different ages compared with non-autistic, and the degree of attentional deficit was related to autism severity (Guimard-Brunault et al., 2013). Similarly, a compromise of visual attention and saccadic reaction to faces and objects was found in a population of high risk 7 month-old infants who later developed ASD. The authors conclude that an *atypical visual orienting* may represent an early manifestation of ASD (Elison et al., 2013). Oculomotor abnormalities as *unusual visual search or saccade production* have been also proposed as the basis of abnormal socio-communicative development (Johnson et al., 2012).

##### Disengagement and ASD

In an extensive review on the impact of attention on core ASD symptoms and cognitive visual strengths, Keehn et al. (2013) highlight that previous reports of atypicalities on either the alerting, orienting or executive attention interacting systems could be secondary to a primary deficit in *disengaging* attention. This is in consonance with abnormal disengagement observed as the earliest symptom in ASD infants (Elsabbagh et al., 2013; Sacrey et al., 2014) and with ERP studies in adults with ASD that showed abnormalities in attention disengagement (Kawakubo et al., 2007).

##### Multiple attention and ASD

At present, most prevailing autism theories are based on deficits on *simultaneous multiple attention* and they state that in order to disclose it, real life evaluation settings should be used (Murray et al., 2005; Fletcher-Watson et al., 2009; Guillon et al., 2014; Jaworski and Eigsti, 2015; Keehn and Joseph, 2016; Unruh et al., 2016; Bolis and Schilbach, 2017). A subgroup of these theories point to deficits in *Simultaneous Attention to the Self and Others* to allow *joint attention* (Mundy and Newell, 2007; Just et al., 2014; Skorich et al., 2017).

##### Summary of ASD theories and its implications with the SC (see Table 1)

**Table 1:** Correspondence between seminal symptoms of each ASD proposed pathogenesis and SC functions^∗^ (see text for references).

ASD proposed theory	Endophenotype/Seminal symptom	Possible SC function compromised
Magnocellular visual pathway dysfunction.	Compromise on visual attention and/or compromise on motion perception and face processing.	Main (or exclusive) gate of the Magnocellular Visual Pathway.
Local over global visual non-conscious (WCC-EPF Model).	Automatic *bias* to local instead of global visual processing.	Exclusive structure described for automatic *bias* for global visual processing.
Innate social motivation compromise.	Emotional hypoarousal or hyperarousal to social stimuli.	First line experience-expectant sentinel structure that triggers the “social brain network.”
Abnormal emotional regulation.	Compromise on the alarm system or emotion system to social and non-social detection and reaction	Detector System and gate to trigger the emotional brain networks (e.g., fight or flight reactions, fear to snakes, etc.)
Face processing abnormalities (emotional or non-emotional processing).	Abnormalities in subcortical face detection network (SC/Pul/Amy).	First line specific neurons with shorter latencies for face recognition (25-ms).
Abnormal attention to social *and* non-social stimuli.	Atypical visual orienting to faces and objects.	First Sentinel/reactive system to novelty (biologic or non-biologic).
	Attention *disengagement*.	Neurons exclusively prepared to *disengagement* functions.
	Compromise of simultaneous or multiple attention.	Elected structure to explain human multiple attention in real word scenes (e.g., MASC model).
Mirror neuron system dysfunction. Abnormal motor and social synchrony (imitation). Compromise on the representation of the *Self.*	Compromise on visual implicit attention that activates the sensory-motor- emotional networks. Simultaneous Attention to the *Self* and *others* to allow joint attention (it also requires simultaneous synchrony between sensory, motor, cognitive, emotional and autonomic variables).	SC functions allow visual implicit attention, non-conscious decisions, visuomotor transformation and simultaneous triggering of sensory-motor-emotional networks. It is the main structure involved on the on each developmental stage of the representation of the *Self.*
Abnormal eye movements and saccade production.	Idem (abnormal saccades).	Main hub for visual search and saccade movements.
Abnormal multisensory integration.	Abnormal visual, auditory and somatosensory integration.	Sentinel multisensory single neurons that integrate visual, auditory and somatosensory input.
Intense world theory	Diminished prepulse inhibition (PPI)	Diminished PPI is exclusively associated with SC compromise.


*^∗^The authors of each theory proposed that the full clinical ASD picture is secondary to the compromise of the seminal symptom described. However the great diversity of ASD symptoms and the high presence of associated disorders could only be explained by a compromise of a hub with sensory, motor, social, emotional, autonomic, communicative integrate functions.*

The previous theories reveal an attempt to disclose a single endophenotype responsible for the clinical manifestations of ASD. Although each proposal is different – magnocellular dysfunction, local over a global processing bias, difficulty to pay attention to multiple stimuli or to a novel stimulus, abnormalities in disengaging attention, lack of social motivation to attend to faces or biologic movement, or abnormalities in saccadic movements- *there is one common factor in all these theories: the automatic or non-conscious visual attention is atypical in ASD individuals*.

The finding of a unified cognitive endophenotype leads to the search of a dysfunction of a specific brain structure. The complexity of the attentional process makes this task difficult; several circuits could be involved. Even the brain’s default network, which is selectively activated by internal mentation, has been proposed as responsible for ASD symptoms (Andrews-Hanna, 2012). Additionally, exclusive cognitive dimensions explain neither the full clinical syndrome nor the comorbidities (dyspraxia, anxiety, emotional abnormalities, sleep disorders, autonomic symptoms, epilepsy, etc.).

**FIGURE 3:**
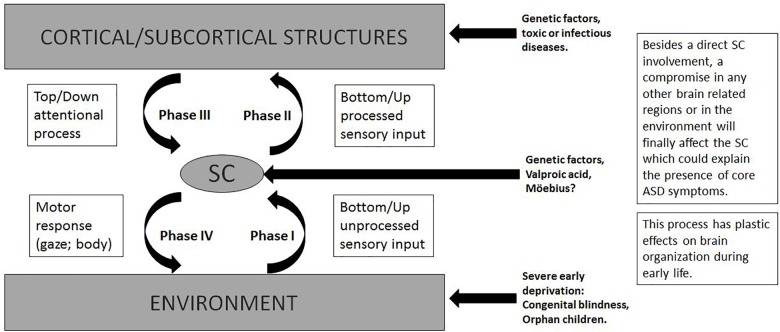
The SC is in the interface of bottom–up/top–down attentional process. Relevant information is sent to higher structures. Motor responses could be conscious or non conscious, limited to eye movement (redirection of the attention) or all body goal oriented response.

The complexity of SC and its influence on the developing brain seem to explain every previous theory and level of ASD compromise. Figure 3 shows the central role of the SC in different phases of these interactions and the impact of different etiologies that can disrupt each step resulting in ASD. A summary of its complex functions is necessary to understand the present theory.

## The SC as a “HUB”

The SC is a very ancient structure correspondent to the optic tectum (OT) in lower vertebrates (amphibians and fish). In mammals, it has a *significant influence on early synaptogenesis* and on plastic reorganization of higher structures after an injury in adults (Rushmore, 2006; Takaura et al., 2011). It contains seven layers that can be functionally and anatomically divided into two parts: The SCs (layers I-III) exclusively *visual* and SCid (layers IV-VII) with *multisensory* and *motor* functions. The “cortex like” anatomic organization (May, 2006), the direct or indirect connections with *most* other parts of the brain, and the strategic localization as a first line structure to environmental (visual/auditory) and body sensory input renders the SC in a unique center implicated in qualitative functions not described on any other brain region (Wurtz and Albano, 1980; May, 2006; Field et al., 2008; Cerkevich et al., 2014). It is a central structure of most subcortical Brainstem/Basal Ganglia loops which are the foundation of higher Thalamic/Cortical Networks (Redgrave, 2010).

Visual information recorded at the SCs is sent through rich connections to the SCid. This small center is perhaps the most integrative area of the brain. It transforms multisensory information and emotions in complex motor commands: gaze movements, head and body turning (Gandhi and Katnani, 2011), arm goal directed movements (Philipp and Hoffmann, 2014) and complex primitive reactions like “freezing,” “hunting,” or “approach” (Furigo et al., 2010; Comoli et al., 2012).

### The Sprague Effect

The SC was usually considered just a passive responder of frontal/parietal orders to produce eye movements. This misconception changed after the description of the “Sprague effect,” a *hemineglect* syndrome produced by a SC unilateral lesion in cats (Sprague and Meikle, 1965; Sprague, 1966) or the resolution of the hemineglect in a patient with a frontal lesion after the inactivation of the contralateral SC (Weddell, 2004) showing the significant influence of the SC on hemispheric functions.

The confrontation of the *blindsight phenomenon* with *the Sprague effect* highlights the silent but crucial functions of the SC. In blindsight the primary visual cortex is compromised but the SC and the magnocellular visual pathway are spared and the individual is able to react to stimuli. Conversely, the Sprague effect results in a severe neglect and makes the individual unable to react, even when the LGN and the primary visual cortex are not compromised. This is an evidence of the role of SC in the continuous, non-conscious multi-attention, emotional processing, including autonomic and motor reactions.

### The Role of the SC on Gaze and Attention

At every moment we draw our attention to different targets and the most conspicuous behavior of our attentional interest is gaze direction. Non-conscious or conscious, driven by extrinsic motivations to promote survival or by intrinsic motivation guided by the pleasure to learn new skills (Caligiore et al., 2015), a simple gaze shift is always the result of a complex mechanism. The SC actively participates in each level of these processes (Corrigan et al., 2015). Every cortical and subcortical region involved in eye movements connect directly or indirectly with the SC (Merker, 2013).

The “MASC” -Model of Attention *in the SC*- proposed to understand multiattentional human tasks is based on real world scenes, combining computational and behavioral neural responses (Adeli et al., 2017). *It seems to be the best suited physiologic model to explain the new theories of ASD based on compromise on simultaneous multiple attention* (see above).

### SC Inner Functions

#### First Sentinel/Reactive System

Animal and human studies demonstrated that the SC has the intrinsic ability to recognize figure configuration (Girman and Lund, 2007; Georgy et al., 2016), to follow an object in movement (Dash et al., 2015; Inayat et al., 2015), and to *select* a new target (Phongphanphanee et al., 2014; Taouali et al., 2015). In rats, the inactivation of the primary visual cortex only *increased* the SC response to moving objects (Zhao et al., 2014). Pre-attentive discrimination between two relevant visual stimuli is accomplished exclusively at the SC (Krauzlis et al., 2013).

Also relevant to ASD theories is the finding in mammals of several groups of specialized SC cells, for example, *neurons in visual layers with the shortest latencies throughout the brain to respond to faces* (25-ms) (Nguyen et al., 2014); *Visuomotor* neurons in intermediary layers (Krauzlis et al., 2013) that allow the enigmatic transformation of visual images into motor commands; *Multisensory* neurons, specially trained to respond to space-temporal congruent stimuli (i.e., biologic events) (Stein et al., 2009); and *Disengagement* neurons, essential to change focus (Ngan et al., 2015). These findings strongly suggest that the SC is highly involved in pivotal operations of attention orienting: recognition of a new relevant clue, disengagement, visuomotor transformation for shifting, and new engagement (de Araujo et al., 2015). Disruptions of *each* of these functions have been linked to ASD pathogenesis (Keehn et al., 2013) (see above and see Table 1).

### SC/Cortex Interactions

The SC functions described above allow only primitive reactions to exogenous stimuli. The existence of Bottom–Up *indirect* multiple connections from SC to the cortex and Top–Down *direct* from the cortex to the SC allow other multiple roles on attentional and goal directed *higher functions*. Animal studies show that postnatal environmental influence shapes the microstructure of the brain through the SC (Rosa Salva et al., 2015) and top–down connections from the cortex prepare special SC neurons as sentinels for future relevant information (Stein et al., 2014). This double input continues to be functional during all life and indicates that *the SC requires both, bottom–up environmental and top-down cortical influence* to function properly.

#### Bottom–Up Indirect SC/Cortical Connections

**FIGURE 4:**
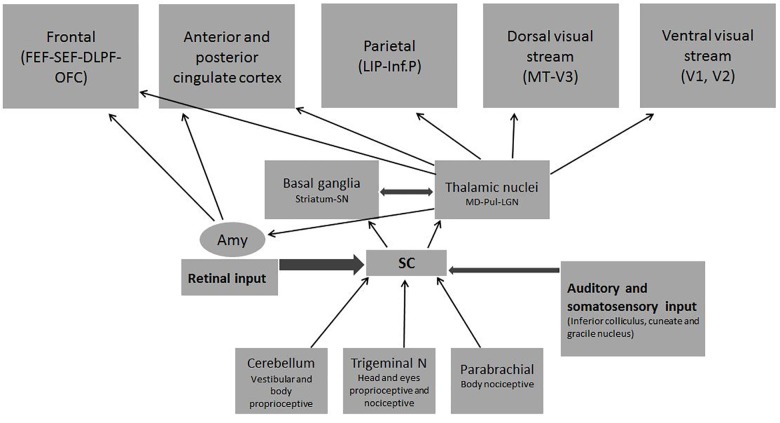
Indirect bottom–up multisensory input from the SC to the cortex: Almost all sensory inputs (visual, auditory, somatosensory, proprioceptive and nociceptive) converge at the Deep SC. The visual input from the superficial SC is the most massive and leads to the correspondence of the rest (somatosensory, auditory, etc.) Several thalamic nuclei receive deep SC input to further reach the cortex.

Somatosensory and motor maps are established inside the SC according to visual input. In turn it organizes parietal and premotor cortical regions through several *multisensory and motor SC/cortical connections* (Figure 4). New evidence indicates that several SC/brainstem/thalamic /cerebellar pathways are essential to cover visual spatial attention (Krauzlis et al., 2013). The present author suggests other more detailed sources for the interested reader (Wurtz and Albano, 1980; Sommer, 2003; May, 2006).

**FIGURE 5:**
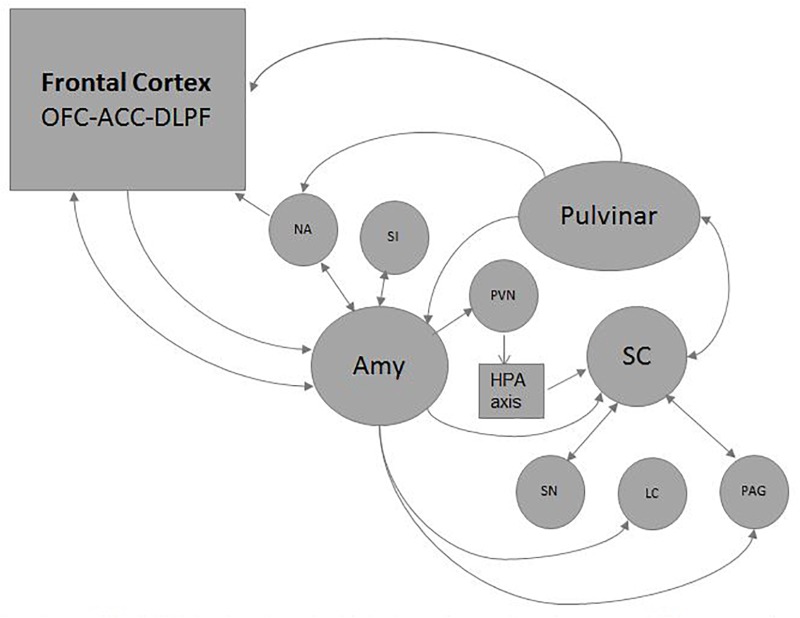
Emotional circuits triggered by the SC: visual and non-visual stimuli carrying emotional content could be processed exclusively by subcortical circuits or to reach the frontal cortex resulting or not in a motor non-conscious or conscious response respectively. The reward dopaminergic system is mediated by the Susbtantia njgra (SN)and the nucleo accubens (NA); the alerting adrenergic system by the locus ceruleous (LC);the cholinergic system by the substantia innomminata (SI). The hypothalamic-pituitary-adrenal axis (HPA) is reached through the paraventricular nucleus (PVN). Some basic emotions like defensive, reproductive, maternal or pain related behaviors are mediated by connections with the periacueductal gray matter (PAG). Through these ways the SC activates simultaneously several attentional, autonomic, endocrine and cognitive functions.

Affective and motivational aspects of visual attention and saccade control activates the SC’ *“Emotion System”* or *“Alarm System”* (Figure 5) (see Vuilleumier, 2015 for a review). It *simultaneously triggers attentional, autonomic, endocrine and cognitive functions* through the *reward* dopaminergic system, the *alerting* adrenergic system, the *cholinergic* system, and the *endocrine* system (Liddell et al., 2005; May et al., 2009; Redgrave, 2010; Tamietto and de Gelder, 2010; Van den Stock et al., 2011; Almeida et al., 2015). Some basic emotions like innate fear to snakes are mediated by SC/brainstem connections without the participation of the Amy (Merker, 2007; Soares et al., 2017). The Pul and the Amy, also considered crucial *Hubs* for emotional and social-communicative development, are activated by the SC before reaching cortical structures (Bickart et al., 2014; Bridge et al., 2015; Diano et al., 2017) (see Figures 4, 5).

Multisensory/motor networks that process imitation abilities conform the “mirror neuron system,” which was also postulated as responsible for ASD pathogenesis (Williams J.H.G. et al., 2001). The mirror system is mainly influenced by emotional and motivational aspects of social and goal directed learning triggered by visual implicit attention (Vivanti and Rogers, 2014). As it was remarked, the SC seems to be the only sentinel structure prepared to trigger automatically and simultaneously sensory/motor/emotional networks.

#### Top/Down Direct Cortical/SC Connections

**FIGURE 6:**
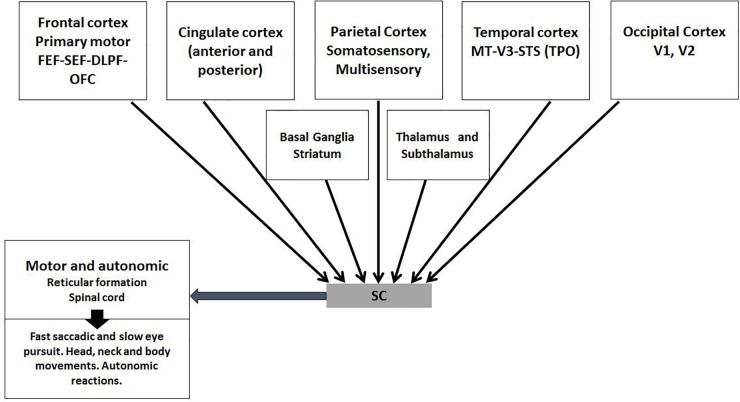
Top–down direct multisensory, motor, and emotion related input from the cortex to the SC. The SC receives direct cortical input mainly from V layer that concentrates all elaborated sensory-motor information from most cortical neurons. Temporal-Parietoccipital (TPO) associated area and frontal lobe regions -premotor mirror networks, emotional networks (OFC) and eye movements areas (FEF-SEF)-should be integrated for mentalize abilities. With this direct input, the SC functions as a contiguous cortical layer. Subcortical nuclei process non-conscious sensory-motor functions and are essential to SC functions.

The SC receives back direct input from several visual and multimodal sensitive and motor cortical regions (Figure 6) (May, 2006). These connections are the most complex known model of top–down influence. During early life it has plastic effects. For example, it has been proved in newborn cats that SC neurons only *become multisensory* after few months of the double training from the environment and the multisensory cortex. These neurons are not only sentinels for very subtle space-temporal congruent events, they also send motor information (Alvarado et al., 2008; Stein et al., 2009; Stein et al., 2014). This sensory/motor integration could explain the attention to the mouth and the imitative movements that infants display in early life, apparently crucial to phonologic discrimination and its influence on language development (Lewkowicz and Hansen-Tift, 2012; Skipper, 2014; Kuhl, 2015).

*Endogenous attention* also activates the SC by top–down influence (Katyal and Ress, 2014) even in absence of the primary visual cortex (Yoshida et al., 2017). Monkey studies suggest that the interaction between vision, memory, self- motion and top–down spatial attention to conscious and non-conscious tracking of multiple visual cues during eye movements is coordinated by the SC (Dash et al., 2016). These functions are essential for mental time travel, for imaginative play and language development (Corballis, 2010). A poor imaginative play and abnormalities in understanding and using language referred to distant facts in time or space are frequently present even in high functioning ASD preschoolers (Rapin and Allen, 1983).

The input from the cortex to the SC is provided mainly by the pyramidal motor cells of the V layer that concentrate all the elaborate sensory-motor information of most cortical neurons. *Although anatomically distant, the direct SC-cortex connections are functionally similar to connections between contiguous cortical layers* (Merker, 2013).

### The SC: A Decision Making Structure

Inside the SC there is a struggle of different forces of incoming stimuli before the selection of an attentional target (Mysore and Knudsen, 2011; Furlan et al., 2015; Inayat et al., 2015; Pretegiani et al., 2015; Wolf et al., 2015; Jagadisan and Gandhi, 2016). The exact mechanism of this competition is a subject of active investigation in bio-cybernetic to understand *how* a visual stimulus is transformed into a response (Brandt et al., 2012; Mysore and Knudsen, 2014; Phongphanphanee et al., 2014; Taouali et al., 2015; Adeli et al., 2017; Veale et al., 2017). This internal activity is influenced by extracollicular input process (Merker, 2007; Thurat et al., 2015; Khan et al., 2016).

The SC follows top–down orders from higher structures (Bauer et al., 2015; Paneri and Gregoriou, 2017) but it is prepared to take the control of any event which could be a source of interest or a potential threat (Maior et al., 2011; Mysore and Knudsen, 2011; Krauzlis et al., 2013, 2014; Phongphanphanee et al., 2014; Schröder and Carandini, 2014). The reaction is not always limited to a gaze change (Lovejoy and Krauzlis, 2010); sometimes a whole body, very fast non-conscious lifesaving reaction is needed and the SC must take the control. In this perspective *SC decisions are superordinate even to cortex decision* (Merker, 2007). Thus, we can suggest that the attentional decisions of the infant influenced by emotional and cognitive dimensions operate through the SC. Then, it is plausible to theorize that the most proposed endophenotype of ASD –the automatic attentional *bias*- is determined by the SC.

### The SC and the Representation of the Self

Different levels of complexity of brain activity have been proposed to explain basic to sophisticated representations of the self (Merker, 2007; Fabbro et al., 2015). Apparently, the *primary self* is sustained purely by a subcortical system (Fabbro et al., 2015) called *the “optic brain”* (Merker, 2007). This conglomeration of nuclei, *whose center is the SC*, concentrates complex innate behaviors: the hypothalamus drives exploratory, ingestive, aggressive, defensive, social, sexual, and parenting basic goal directed behaviors and the PAG is involved in pain and powerful emotional reactions (Merker, 2007). These subcortical nuclei seem to be essential for further development of reflective knowledge based on *self-consciousness*, sustained by frontal, parietal and temporal structures. With the contribution of these cortical networks, babies start to recognize themselves in a mirror at 18 months of age. Few months later (∼24 months) they achieve the most complex representation of the self, exclusive of humans *-the narrative-self-*, supported by three cognitive aspects: (1) the ability to mental time travel to reconstruct past events and imagine possible future scenarios, (2) mentalizing abilities (theory of mind) and (3) language abilities that allow sharing it with other congeners (Fabbro et al., 2015).

The functional link between widespread bilateral cortical regions required to sustain this complex function is based on synchronized gamma band (∼40 Hz) neuronal activity which is highly coordinated with activity recorded at the SC (Fabbro et al., 2015) and the Pul (Soares et al., 2017). Recent MEG studies in humans support the existence of an impaired gamma band synchrony during eye-gaze processing in ASD (Richard et al., 2013). A direct relationship has been found in the severity of ASD and the compromise of shared attention secondary to a poor self-categorization (Skorich et al., 2017).

### The SC and the Building of the Social Brain

The newborn’s ability to follow biologic motion and faces (Greenough et al., 1987) is sustained by *experience-expectant* circuits. Apparently, similar to the early *imprinting* phenomenon on birds, this event triggers simultaneously neurotransmitters and genes inducing qualitative changes on several brain regions responsible for the future social and cognitive style of the individual (Klin et al., 2015; Rosa Salva et al., 2015). The SC ascending connections are the candidate for an initial *plastic* effect on more *“experience-dependent”* higher circuits of the social brain. Prenatal and early postnatal animal studies have shown that the first neurotransmitter responsible for synaptic prune and specialization is GABA (Hensch, 2016). Evidence shows that the *SC contains the largest amounts of GABA and the highest density of GABAergic synaptic terminals in the brain* (Grantyn et al., 2011). Alterations in early GABA expression secondary to gene defects, perinatal trauma or toxic effects during pregnancy could result in neurodevelopmental disorders (ASDs, Rett Syndrome) and/or epilepsy (Abrahams and Geschwind, 2008; Takesian and Hensch, 2013), and repetitive circling behavior in rats (Velíšek et al., 2005). Not only does GABA play a role in early brain organization, but also almost all neurotransmitters (including nitric oxide) are involved in SC functions indicating its ubiquitous influence on several brain structures necessary to accomplish complex functions (Fuentes-Santamaria et al., 2008).

### Effects of SC Lesions on Behavior in Humans and Animals

Few descriptions of motor or saccadic eye movement secondary to isolated unilateral SC lesion in adults were found in the literature (Heywood and Ratcliff, 1975; Girotti et al., 1979; Paidakakos et al., 2012).

More than a half century ago Denny-Brow described behavior abnormalities in monkeys and in a 17 year old girl with SC lesion. The author was impressed by how this small area inactivates the elaborate hemispheric organization for reaction to the external world. At that time he proposed that the SC was essential for social development (Denny-Brown, 1962).

#### Animal Studies

Bilateral lesions of the SC in infant capuchin monkeys impaired fear of snakes (Maior et al., 2011) and, on follow-up, they showed a transitory compromise of social behaviors. The authors proposed that *the SC may play a key role in early stages of social development*, but after this period its neural substrate involves larger networks (Maior et al., 2012). Despite the fact that the SC has connection with all brain areas involved in gaze direction, its ablation in rats does not result in absence of eye movements. Instead, it severely affects the ability to *select* relevant information to survival (ex.: to respond to a threat) and to establish *priorities* from several clues in covert attention to decide *where* the next gaze should be oriented (Merker, 2013) (which is an endophenotype of ASD) (see Table 1).

## Beyond Social/Communicative Compromise: Stereotypies and Motor Abnormalities

Based on the ubiquitous presence of hyper-reactivity to nociceptive responses, acoustic startle response, and diminished prepulse inhibition (PPI) Markram and Markram (2010) proposed the *“Intense World Theory”* to explain ASD. They point to a prenatal compromise of the brainstem resulting in hyper-functioning of local neural microcircuits. Diminished PPI has been specifically associated with SC compromise (Soares et al., 2017) and it is in consonance with its extremely excitatory role that needs continuous gabaergic inhibition from several surrounding nuclei, intrinsic interneurons (Merker, 2007) and the cortex (Yu et al., 2013). Prenatal exposure to valproic acid in humans results in *Fetal Valproate syndrome*, associated with a high ASD prevalence (Williams G. et al., 2001; Schneider and Przewłocki, 2005) and a rodent model of autism with striking behavioral, anatomical and pathological, similarities was created by exposure of rat fetuses to *valproic acid (VPA)* (Schneider and Przewłocki, 2005). This is in concordance with the specific compromise of *gabaergic SC’ neurons* in rats exposed to the drug that not only showed difficulties in social and exploratory behavior but also abnormal sensory patterns of hypo or hyper-reactivity to nociceptive responses, to acoustic startle response, and diminished PPI (Dendrinos et al., 2011; Nakamura et al., 2015). Similarly, *increased seizure activity* secondary to hyperconnected networks in the OT has been found in tadpole prenatally exposed to valproic acid (James et al., 2015). Less circumscribed (not limited to the SC) prenatal focal brainstem lesions that result in *Möebius syndrome* or *Thalidomide syndrome* were also associated with high ASD prevalence, abnormalities in sensory processing, and epilepsy (Miller et al., 2005; Miller and Strömland, 2011).

All previous listed findings seem to be in consonance with brain abnormalities observed in ASD individuals such as increased local subcortical hyperconnection and abnormalities of early pre and post-natal synaptic organization (Abrahams and Geschwind, 2008; Markram and Markram, 2010).

*Motor abnormalities in synchrony and imitation* are among the most common earlier ASD’ manifestations (Marsh et al., 2013) and have been attributed to abnormal brain connectivity (Fishman et al., 2015) or magnocellular dysfunction (Ronconi et al., 2012). *Social synchrony* development is influenced by several variables like innate motivation and emotions, mimetic desire, visuomotor integration, implicit and explicit attention (Fitzpatrick et al., 2017). As it was previously exposed, the SC has a central role in the integration of sensory, motor, and emotional variables, necessary for mentalizing functions and the representation of the self.

## ASD Etiologies, Timing of Clinical Manifestation and Neurodevelopmental Course

Non-defined genetic causes represent the most prevalent etiology of ASD and several hundred of genetic markers have been proposed (Willsey et al., 2013). This complexity is increased by prenatal and postnatal genes-environment interactions (Fein and Helt, 2017). For that reason, this line of research could be useful to recognize subtypes of autisms but unfruitful to explain the presence of the cluster of symptoms in the entire spectrum including children with non-genetic etiologies. It is more rational to propose that multiple etiologies confluence on specific networks in a specific age window, provoking a dysfunction responsible for the myriad of core symptoms (see Figure 1). In this section, etiologic genetic and non-genetics factors will be discussed under the perspective of prenatal and early postnatal SC involvement.

### Prenatal Etiologies

It is clear that *experience-expectant* subcortical SC circuits that drive social motivation are almost exclusively influenced by genetic aspects. It is probable that a single compromise of any gen responsible for diverse interacting substances like oxytocin (OXT), dopamine, GABA, glutamine, acetylcholine, endogenous opioid, serotonin, etc., could jeopardize the prenatal emergence of social motivation.

Two conspicuous examples are, on one side, at the very beginning of the pathway, the finding of autism-relevant social abnormalities in *Engrailed-2* knockout mice, a gene responsible for *retino-tectal (SC) axon guidance* and for proper development of monoaminergic hindbrain circuits (Brielmaier et al., 2012) and on the final top–down influence, the results of a multidisciplinary study revealing that several ASD associated genes converge over midfetal layer 5/6 cortical projection neurons (Willsey et al., 2013) which, from every cortical lobe, *directly* connect with the SC (Merker, 2013).

There are other possible mechanisms, to name a few due to the complexity of structures and functions involved. For example, a single gene defect compromising a peptide like OXT or its receptors have been linked to a reduced ability to face recognition, face emotion, or direction of gaze detection in members of families with an autistic child (Skuse et al., 2014). OXT was specifically linked with the SC in monkeys (Freeman et al., 2014) and, fMRI images in humans showed Amy and SC activation with enhanced attention to faces by OXT administration (Gamer et al., 2010). *Serotonin* has been highly implicated in abnormal emotional regulation, stereotypies, and obsessive symptoms of ASD individuals (Tuchman, 2006; Johnston, 2011). Serotonin transporter gen markers were linked to anatomic and functional abnormalities in a specific emotion related SC-Pul-Amy pathway activated by eye contact and, apparently essential to the development of social cognition (Skuse, 2006). In rodents, the influence of the SC on cortical serotonin regulation in response to visual and non-visual stimuli has been shown (Dringenberg et al., 2003; Bezdudnaya and Castro-Alamancos, 2014). An fMRI study showed bilateral dysfunction of dopaminergic networks during reward related social games (Assaf et al., 2013). Genetic studies pointed to abnormalities in dopaminergic receptors involved in the rewarding value of joint attention in ASD’ individuals (Gangi et al., 2016); several dopamine networks are activated by the SC (see above for references). Most of these genetic mechanisms are still unknown, which could explain the frequent non-defined genetic etiologies that result in ASD.

Prenatal neuroinflammation is also a frequently proposed etiology of ASD (Bilbo et al., 2018; Ohja et al., 2018). A selective compromise of social behavior mediated by the OT was observed in tadpoles exposed to elevated levels of *pro-inflammatory cytokines (PIC)* during early development (Lee et al., 2010). Additionally, it has been previously described that non-genetic prenatal conditions like fetal valproic acid or thalidomide syndrome directly compromise the SC (see above).

### SC/Cortex Postnatal Mutual Influence

The postnatal appearance of the symptoms after 2 months of normal eye gaze in some children (Jones and Klin, 2013) or the occurrence of an autistic regression between 1 and 3 years old in almost a third of ASD’ children is suggestive of an active postnatal pathogenic compromise. If early interactions between environment and the genes shape several brain circuits and the “bottleneck” or the “interphase” between both is the SC, any significant genetic or non-genetic etiologic event will impact directly or indirectly on it. The postnatal complexity of environmental-gene interactions increases the vulnerability for pathogenic factors, mainly on genes that participate in neurotransmitter physiology, and brain microstructure. Most of the genetic markers of ASD are related to synaptic formation (Abrahams and Geschwind, 2008; Adviento et al., 2014; Sala et al., 2015; Sanders et al., 2015), cell adhesion molecules (Klin et al., 2015), axon guidance (Suda et al., 2011) or the metabolism of several neurotransmitters including GABA (Grantyn et al., 2011) or oxide nitric (Campbell et al., 2013; Sweeten et al., 2004).

It is worth noting that the synapses are diffuse but their compromise in ASD affects selectively some circuits, sparing or even enhancing others. This observation strengthens the theory of a lack of visual imprinting provided by the magnocellular pathway through the SC. Neuroanatomical and neurofunctional imaging studies disclose a *selective* compromise of networks that supports faces and motion perception, and emotional processing (Patriquin et al., 2016). White matter tract studies reveal hypo-connectivity of the large-scale tracts (Courchesne et al., 2007), more specifically the bilateral uncinate and superior longitudinal fasciculi (Jou et al., 2011). All these networks support social and communicative development and are activated by the SC after birth.

The compromise of brain connectivity is not limited to the cortex. A large scale multidisciplinary evaluation of diverse images studies not only confirmed the selective long range cortico-cortical hypoconnectivity in areas related to the social brain, but also highlighted *a new finding*: hyperconnectivity in subcortical thalamic structures (Di Martino et al., 2014) This is in consonance with the animal models of ASD prenatally exposed to valproic acid that also showed abnormal response to sensory stimuli and stereotypies (see above for references). Cortical top-down direct connections exert mostly inhibitory effects on SC neurons (Yu et al., 2013). Hence, it is possible that genetic and non-genetic factors which compromise distant but connected regions with the SC including the cortex could be a potential source of ASD pathogenesis; for example: Tuberous sclerosis, West Syndrome or electrical status epilepticus during slow sleep (McVicar et al., 2005; Tuchman et al., 2009).

### Non-genetic Postnatal Environmental Factors

A high prevalence of autistic behavior, including repetitive and restricted range of interest, was found in orphan children with severe early deprivation and in adopted children after early care breakdown. The main determinant factor was the occurrence of the deprivation during the first 6 months of life or longer (Rutter et al., 1999; Kreppner et al., 2010; Nelson et al., 2011; Green et al., 2016). Diverse functional and anatomic brain studies on severely deprived orphans showed abnormalities in the Amy, the hippocampus, the ventral striatum, the orbitofrontal cortex and reduced white matter organization of the bilateral uncinate and superior longitudinal fasciculi (Nelson et al., 2011). These findings also support the existence of a *critical period* that needs the contribution of the environment to produce the synaptic and neural changes necessary for the development of the social networks. This could explain the very high prevalence of autism regression between 15 and 30 months of age, even in children with CB that showed a visual improvement after the first year of life (Dale, 2005) and the significant difference of ASD in children with CB compared with partial or acquired blindness (Hobson and Bishop, 2003; Jure et al., 2016).

## Developmental Trajectories

Based on the interplay of “social motivation” and “social cognition,” subserved by “experience-expectant” and “experience-dependent” brain circuits respectively, this model could also explain the different developmental trajectories described in ASD children (Fountain et al., 2012). During the first months of life, “experience-dependent” higher circuits are modeled by “experience-expectant” networks. A change of this dominant role in the opposite way (i.e., cortical over subcortical structures) from the first months through adulthood seems to occur gradually. The adult phenotype seems to be the result of both: the intensity of ASD’s initial symptoms and the late appearing cognitive resources fed by cultural influence. *Autism recovery* or *optimal outcome* has been described in a minority of sighted individuals (∼20%) after 8 years of age, with mild ASD before 5 years old and above average level of cognition. The authors argued that this compensation could be secondary to the use of explicit, higher order, resources to substitute weak implicit social processing (Fein et al., 2013). The most dramatic *autism recovery* observed in an important minority of children with CB (Hobson and Lee, 2010) could be explained by a presumed normal genetic background for social abilities and a great need of verbal communication regarding its unique role as a vehicle for learning in blind individuals. On the other hand, the very high prevalence of *Autism regression* in CB before 3 years of age (Jure et al., 2016) could be the result of a secondary degeneration (diaschisis) of selective circuits as a result of a total lack of visual input during the first months of life.

## Associated Language Disorders and Intellectual Deficit (ID)

Regarding that most of the numerous genes involved in the pathogenesis of ASD are related with early axon, synapsis formation and neurotransmitters, the most probable scenario is that different groups of those genes participate in different developmental stages of brain organization and function. If the genetic compromise affects the connections diffusely, the most likely clinical result will be the association of ASD with ID (which is present in ∼50% of ASD individuals) and more severe language disorders. If genes with a more selective function are affected, i.e., some of those that are activated by the SC in the postnatal period, the clinical manifestations will be more selective, and perhaps limited to ASD symptoms without intellectual compromise.

Additionally, the SC houses several types of neurons, dependent of several unknown genetic markers (Byun et al., 2015) that could be selectively affected resulting in different ASD phenotypes. For example, hypothetically, a compromise of multisensory SC neurons will result in less extended multisensory cortex and more cortical neurons recruited to both, isolated auditory and visual sensory processing respectively. This could explain not only most of ASD symptoms (including language disorders) according to some authors (Stein et al., 2014; Stevenson et al., 2014) but also the higher ability to visual local processing and the higher frequency of absolute pitch in ASD population (Heaton et al., 2008). A dysfunction of neurons that process facial configuration could explain the attentional tendencies to objects instead of congeners in early life. On the other hand, the rigidity, narrow interest and selectivity for some particular topics could be secondary to a compromise on disengagement neurons.

To understand the influence of attention on language development it is essential to clarify the variability in the attentional compromise: attentional neglect as in “Sprague effect” means that the individual is unable to visualize or to process the presented information despite all the efforts made by the interlocutor (e.g., to share attention over a proposed activity); this severe neglect from infancy could result in a complete lack of language development. Children with a selective attentional interest, e.g., on numbers, letters, songs, or TV programs can be able to repeat the alphabet, several songs with excellent phonology, or numbers in two different languages, but will display a serious compromise in communication. A mild compromise, limited to multiattentional tasks, can explain the ability of some high functioning ASD individuals to sustain a formal dialog with one or two persons but the inability (or avoidance) to do it informally, in a group of several individuals. As it was previously mentioned, new evidence points to abnormal social attention to explain the language compromise on children with ASD (Tenenbaum et al., 2014).

## Summary and Conclusion

This review on two parallel lines of research, ASD pathogenesis and SC functions, discloses several common points worth investigating (see Table 1). The three levels of ASD’ compromise (clinical, etiological and pathogenic) could be explained by the role of the SC as a *hub* for developing brain networks that support social, emotional and communicative behaviors (see Figures 1, 3). Genetic and epigenetic and non-genetic prenatal or postnatal etiologies can affect the role of SC to transform early visual experiences into microstructural cerebral changes. Different pathogenic lines compromising the SC and affecting neuronal differentiation, synaptic formation, pruning, neurotransmitters, neurotrophic factors, or neurohormones (see text above and see Table 2) could explain the diversity of clinical subgroups. The timing of postnatal biological disruption parallels the timing of clinical manifestation. Most ASD cognitive theories, instead of being rejected, seem to adequately fit in this framework to explain all ASD symptoms in one individual and qualitative differences between subgroups. Every seminal symptom or endophenotype of each previous proposed theory is explained by a disruption of one of the several SC functions (see Table 1). Only the integration in this very small structure of all orienting senses with attentional, autonomic, endocrine, emotional, motivational variables and its transformation on motor commands, could explain the repetition of such variety of symptoms -social, communicative, autonomic, sensory-motor, emotional, etc.- in almost each individual with the disorder. Most evidence shows that this simultaneous integration led by vision and its influence on several cortical and subcortical structures (by gamma band activity?) is necessary to accomplish the synchrony needed to multiple attention of several variables required on real life social environments (see above for references). The interplay of a continuum set of abilities, or deficits, of the two broad cognitive styles: those provided by the SC on one side -non-conscious, peripheral, automatic, simultaneous, global-, and the more focal, conscious, analytic and detailed system -independent of the SC- on the other side, could explain the broad spectrum of the clinical compromise and the uneven skills usually observed in ASD individuals. The lack of the inhibitory action of cortical structures over the SC seems to be the best explanation to the abnormal response of sensory stimuli, the frequently associated anxiety disorders, obsessive thinking and the stereotypies.

**Table 2:** Etiologies and brain mechanism proposed for ASD and its coincidence with SC compromise (see text for references).

Etiologies or brain mechanism of ASD	SC functions
Genetic markers of ASD are related to synaptic formation, cell adhesion molecules, axon guidance, etc.	The SC is the main brain organizer (by early visual input) during the first months of life. Several specific genes related with retino-tectal axon guidance and cortical-SC connections are related with ASD etiology.
GABA is the main synaptogenic neurotransmitter during early life.	The SC is the main GABAergic brain center with a generalized influence on sensitive, motor and emotional circuits.
Fetal valproate syndrome	Specific compromise of *gabaergic SC’ neurons* in rats exposed to the valproic acid: they showed difficulties in social and exploratory behavior, abnormal sensory patterns and diminished prepulse inhibition (PPI).
Others neurotransmitters and hormones (OXT, testosterone, dopamine, glutamate, nitric oxide, choline, etc.) have been implicated in the pathogenesis of ASD.	Most reward related dopaminergic circuits are activated by the SC. The interaction between OXT and testosterone has an influence on the gaze behavior toward faces (aversion or indifference)^∗^ which are closely related with the SC. Most neurotransmitters and neurotrophic factors have input and/or output influence on the SC.
Impaired gamma band synchrony in ASD.	Widespread cortical synchronized gamma band activity is triggered by SC activation.
New images studies found selective long range cortico-cortical hypoconnectivity in areas related to the social brain, and *hyperconnectivity in subcortical* thalamic structures.	The SC could be responsible of the hypoconnectivity of the *social brain network* regarding its specific role on these brain areas during early human development. The recent finding of subcortical hyperconnectivity reinforces the theory of subcortical network compromise (whose center is the SC) and explains several symptoms such as the stereotypies, the abnormal PPI, obsessions, etc.
Prenatal neuroinflammation is a proposed etiology of ASD.	Impaired social behavior by compromise of the *optic tectum* was observed in tadpoles exposed to elevated levels of *pro-inflammatory cytokines (PIC)* during early development.


*^∗^Baron-Cohen (2017).*

The interaction of experience-expectant circuits that drive innate social motivation and late maturing experience-dependent networks that support social cognitive development is the best framework to explain the different neurodevelopmental courses (regression or optimal outcome) and the social subtypes. For example, an innate lack of social motivation would result in an aloof or passive social style. The very frequent social inappropriate subtype could be secondary to a high innate desire of interaction but without the resources (attentional, cognitive, etc.) provided by higher order cognitive processes that require frequent and long-lasting bottom–up and top–down SC-cortical interaction to allow a fully developed social cognition. It could be concluded that it seems unlikely to find another brain structure with the complexity of SC suitable to explain the complexity of all ASD variables.

## Future Research

Regarding the SC anatomic location, its complexity, the plastic changes it suffers during the first months and the limitations for *in vivo* studies in infants, images and functional studies represent a significant challenge. Animal studies are limited due to their lack of higher order social and communicative abilities compared to humans. New technologies are indispensable for revealing the intricate mechanisms that govern the qualitative brain changes after the exposure of visual experiences. However, *it is also necessary to change the focus of research from cortical to subcortical structures regarding its prominent role on hemispheric organization during early life*. A search for anatomic and physiologic differences in the SC of non-autistic *vs.* autistic individuals could provide an initial clue for further research.

While the previous description was centered on the SC, it should be stressed that it is closely joined to the Pul. The inferior and the lateral Pul have a key role in emotional and social functions and are more developed in humans than in other primates. The main questions are: is the SC essential for social and communicative development during the first months of life? Could it be replaced by another structure like the Pul? Which one of the two structures is more relevant in ASD pathogenesis? Is it possible that selective compromises of SC functions could explain different pathogenic ASD pathways? What are the genetic-epigenetic interactions mediated by the SC necessary to support the development of social/communicative behaviors? (For example: “what are the effects of visual experiences and social interactions on the SC during the first 6 months of life to turn on and off different genes that activate brain mechanisms to allow social and communicative development?”). To respond to these questions clinical and biological researchers must work in collaboration.

## Author Contributions

The author, a child neurologist interested in neurodevelopmental disorders, started a review research prompted by his previous work that showed that congenital blindness conveys a huge prevalence of autism. Two parallel lines of research, one on previous pathogenic theories and etiologies of autism, and the second on superior colliculus functions, were confronted to elaborate this hypothesis on autism pathogenesis.

## Conflict of Interest Statement

The author declares that the research was conducted in the absence of any commercial or financial relationships that could be construed as a potential conflict of interest.

## Abbreviations

Amy: amygdala
ASD: autism spectrum disorders or autism
CB: congenital blindness
EPF: enhanced perceptual functioning model
FEF: frontal eye fields
fMRI: functional MRI
IFG: inferior frontal gyrus
LIP: lateral intraparietal area
MD: mediodorsal thalamus
MEG: magnetoencephalography
MT: middle temporal cortex
MRI: magnetic resonance imaging
OXT: oxytocin
PAG: pariacueductal gray matter
PVN: paraventricular nucleus
Pul: pulvinar
SC: superior colliculus
SCs: superficial SC
SCid: intermediate/deep SC
SEF: supplementary eye fields
SN: susbtantia nigra
STS: superior temporal sulcus
TMS: transcranial magnetic stimulation
V1: primary visual cortex
V2-V3-V4: secondary visual cortex
WCC: weak central coherence theory
